# The Living and the Dead

**Published:** 1893-05

**Authors:** Kirke White


### THE LIVING AND THE DEAD.

In New York city, the great metropolis of the new world, I have always found myself remarkably impressed by one peculiar circumstance,—the contrast between the bustling streets, full of living faces, and to-day objects of all kinds, and the quiet and ancient churchyards which are found in the lower part of the city. But a few yards off a busy thoroughfare, which for scores of years has borne the press of breathing men,—where the luxuries and conveniences of life are presented in infinite variety, to attract and fix the attention of the passenger, and where men and women seem so much engaged in the affairs of this world as hardly to be conscious that there is any other, you find the silent and cloistered precinct of the old parish church, paved with the memorials of past generations who once passed as gaily and thoughtlessly among the ways of the city as those you have just seen, but have long retreated to this narrow place, so near, yet so different from all their former haunts. The transition, in your case as a visitor, as well as in their's who pass in this space from life unto death, is the most sudden and rapid that can be imagined, yet how different all the attributes of the two scenes! In the other, how dismal and, in general, how neglected!

HERE you have, at one moment, perhaps the most animated and cheering scene in the world; *there*, at the next instant your gaze is turned upon the most torpid and gloomy. At one twinkle of the eye, you find life and all its affairs exchanged for death and all its circumstances, and pass at a single step, from the lightest to the gravest of reflections. I am not aware of any place where this contrast is presented in a more striking manner than it is from the steeple of Old Trinity Church which rises above the noise and din of the great city. After fluttering a long time through the crowded streets amidst numberless beings to whom death seems the remotest of all ideas, you are led to the church from whence you command a view of the far-spread city, with its spires peering out here and there, to mark the extent of a waste of houses which would otherwise be hardly distinguishable, while close beneath your feet you see the outlines of the huge church surrounded by its extensive cemetery, a city of the living and a city of the dead, being thus brought into immediate comparison and weaving out of their separate influences the most impressive of all lessons.

The place of the living is, as you can see and hear, one of the busiest scenes of men's labors. It contains hundreds of thousands of industrious human beings all toiling on from morn to eve in their various pursuits, some for mere subsistence, others for loftier objects, but all animated by human motives, and, in general, thinking of nothing in the meantime beyond the bounded horizon of mortal life. How many hearts are there bending anxiously over accounts, in which their own welfare and that of all who are dear to them is concerned! What numberless modes are there assumed of gaining that surplus of value called profit, on which so much of the comfort of individuals depends! How keenly are even pennies, in many cases there aimed at and longed for,—what emotions of the soul, what lightnings of the eye, what contentions between man and man there arise from considerations of money, and of the almost infinite benefits which money can purchase!

The whole vast space is covered to its uttermost nook with human creatures whom the common doom has compelled, for the sake of bread and other sublunary enjoyments, to narrow their souls to the affairs of lucre while they every moment tend onward to a fate more glorious or more terrific, than imagination can picture and are even now capable of thoughts and sentiments far above this world.

And all this, too, is only a detachment of that trifling section of the human race, called the present generation. On or near the same ground have men toiled and moiled as anxiously as these for many years; and what *is* it all and what *will* it all come to? To the little fold which we see directly beneath, a space not large enough to contain the lodgings of a hundred living families, but which has received into its bosom thousands after thousands of the more easily accommodated dead and will in time absorb multitudes as great and yet never cry enough. Yes, as the poet sings, "The paths of glory lead but to the grave." That small spot, of which so few are now thinking as they pace the streets of the busy city, is the real termination of all the journeys they are making. Go they east or west, north or south, be business or be pleasure their immediate object, to this dismal scene must they arrive at last.

Not a step do they take which does not bring them nearer to this ultimate point, although it may seem for the time to lead them in a different direction.

Every effort which they are making to exalt themselves in this world only renders them the richer spoil for the daily hecatomb here offered up to death, and in which, sooner or later, they must bear a part. Every improvement which they can make in their circumstances, while they live, gives them but the chance of a more secluded spot in this gathering place of the departed, or a monument which will longer continue to tell its unmeaning and unregarded tale. In a few short years they and all their joys and sorrows, their greatness or their loneliness will have shrunk into this cold and uncomely scene, while their various walks of business and labor are occupied by others to whose pursuits a similar bourne will in time be assigned. It is not, perhaps, to be desired that reflections of this solemn kind should often or permanently fall upon the minds of men; for, if we were to be perpetually brooding over the gloomy view which the end of life presents, we would embitter that life to a degree rendering us quite unfit for the proper management of either our temporal or spiritual concerns. In general, however, human beings, or at least that portion of them called men of the world, are in little danger of suffering from this cause. It is more frequently observed that a constant commerce with the world hardens the heart toward all *beyond* the world, if not also too much *in* the world, regarding which it is desirable that we should keep *our* feelings awake.

It cannot but be salutary, then, for all who are in danger of falling into this insensibility to turn their minds occasionally to the affairs of mortality, and, seeing the uselessness of all acquisitions after death, the vanity of all terrestrial glory and the community of destiny which overhangs the various orders of the human race, open their hearts more freely to the claims of their fellow creatures around them, and otherwise lay up those stores which will stand in good stead when they and the world have alike passed away. KIRKE WHITE.

